# Gold Nanoparticles of Diameter 13 nm Induce Apoptosis in Rabbit Articular Chondrocytes

**DOI:** 10.1186/s11671-016-1461-2

**Published:** 2016-05-13

**Authors:** Hao Huang, Ying-yao Quan, Xiao-ping Wang, Tong-sheng Chen

**Affiliations:** Department of Pain Management, The First Affiliated Hospital of Jinan University, Guangzhou, 510630 China; MOE Key Laboratory of Laser Life Science and College of Biophotonics, South China Normal University, Guangzhou, 510006 China

**Keywords:** Gold nanoparticles, Chondrocyte apoptosis, Size-dependent cytotoxicity, Osteoarthritis

## Abstract

Gold nanoparticles (AuNPs) have been widely used in biomedical science including antiarthritic agents, drug loading, and photothermal therapy. In this report, we studied the effects of AuNPs with diameters of 3, 13, and 45 nm, respectively, on rabbit articular chondrocytes. AuNPs were capped with citrate and their diameter and zeta potential were measured by dynamic light scattering (DLS). Cell viability was evaluated by Cell Counting Kit-8 (CCK-8) assay after the rabbit articular chondrocytes were pre-incubated with 3, 13, and 45 nm AuNPs, respectively, for 24 h. Flow cytometry (FCM) analysis with annexin V/propidium iodide (PI) double staining and fluorescence imaging with Hoechst 33258 staining were used to determine the fashion of AuNPs-induced chondrocyte death. Further, 13 nm AuNPs (2 nM) significantly induced chondrocyte death accompanying apoptotic characteristics including mitochondrial damage, externalization of phosphatidylserine and nuclear concentration. However, 3 nm AuNPs (2 nM) and 45 nm (0.02 nM) AuNPs did not induce cytotoxicity in chondrocytes. Although 13 nm AuNPs (2 nM) increased the intracellular reactive oxygen species (ROS) level, pretreatment with Nacetyl cysteine (NAC), a ROS scavenger, did not prevent the cytotoxicity induced by 13 nm AuNPs, indicating that 13 nm AuNPs (2 nM) induced ROS-independent apoptosis in chondrocytes. These results demonstrate the size-dependent cytotoxicity of AuNPs in chondrocytes, which must be seriously considered when using AuNPs for treatment of osteoarthritis (OA).

## Background

Osteoarthritis (OA) is the most common and disability joint disease [1, 2]. Treatments of OA are divided into non-surgical interventions for patients in early and moderate stages and surgery interventions for patients in advanced stages according to the severity of OA [3–5]. OA therapy was mainly to reduce pain and increase life quality of the OA patient [6]. Oral non-steroidal anti-inflammatory drugs (NSAIDs), the most efficacious and widely used treatments for knee OA, have shown some risks of systemic adverse events including gastrointestinal or cardiovascular abnormalities [7]. In this light, local treatments appear to be preferable especially in older OA patients with comorbidities [7]. Although intra-articular injection of hyaluronic acid may be a viable alternative to NSAIDs for knee OA [7], its clinical use is still controversial [8]. Because of the inefficacy and “not cost-effective” of intra-articular injection of hyaluronic acid, three clinical practice guidelines, including the National Institute for Health and Care Excellence (UK), National Collaborating Centre for Chronic Conditions (UK), and American Academy of Orthopaedic Surgeons (USA), were recommended against the use of intra-articular injection of hyaluronic acid in the treatment of knee OA [8]. In addition, multiple intra-articular injections of hyaluronic acid, which may increase the risk of joint infection, are needed due to the short residence time of hyaluronic acid in joint [9, 10]. It was also reported that intra-articular injection of corticosteroids might increase adverse effects including joint infection, intra-articular and periarticular calcifications, cutaneous atrophy and cutaneous depigmentation when used repeatedly for OA treatment [11]. Therefore, it is currently desired to develop a new clinical methodology for OA treatment.

Gold colloid or gold salts can exert anti-inflammation effect by downregulating the expression of interleukin-1β, prostaglandin E2, and cyclooxygenase-2 [12–15], which plays a crucial role in the pathogenesis of OA [16–21]. Recently, it was reported that mice model of OA, after treatment with gold nanoparticles (AuNPs) combined with curcumin, presented less severe in the histological lesions [22]. Similarly, AuNPs (7–130 nm) capped with different drugs exhibited potential treatment for OA [23–25]. Our recent studies demonstrated that reactive oxygen species (ROS) played a key role in chondrocyte apoptosis that had been reported to be correlated with the severity of OA [26, 27]. AuNPs (~3.5 and ~50 nm) have been demonstrated to be a potential antioxidant [28, 29]. ~64.83 nm AuNPs capped with hyaluronate and tocilizumab showed therapeutic effects on rats with collagen-induced arthritis (CIA) [30]. Similarly, 13 nm AuNPs capped with galectin-1 showed therapeutic effects on CIA rats [31], and 13 nm AuNPs capped with citrate could also ameliorate CIA in rats [15, 32].

**Fig. 1:**
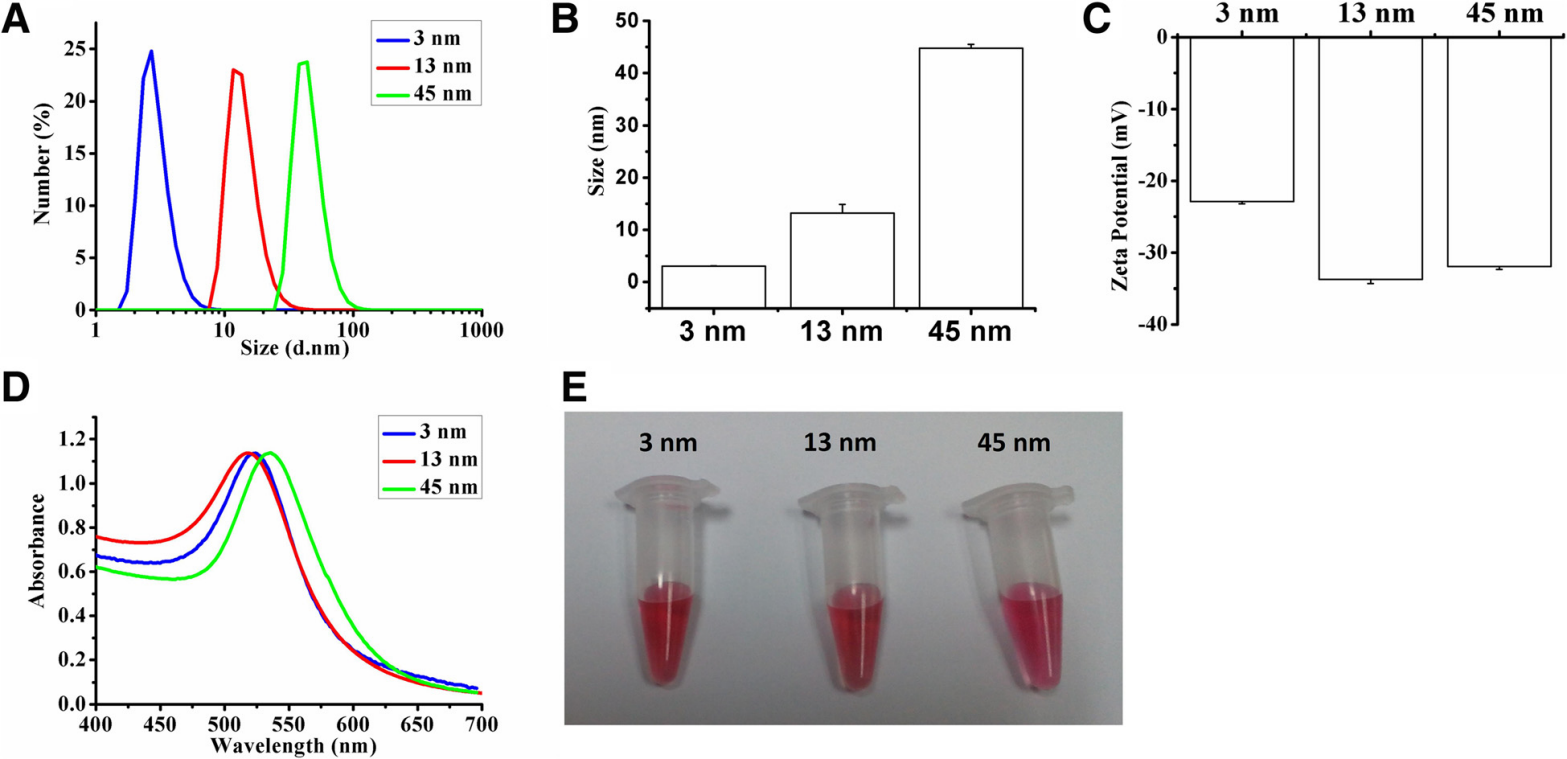
Characterization of AuNPs. **a** The size distribution of 3, 13, and 45 nm AuNPs by number. **b**, **c** The hydrodynamic diameters and zeta potentials of 3, 13, and 45 nm AuNPs (*n* = 3). **d** Photographic images and UV-vis spectra of 3, 13, and 45 nm AuNPs. **e** Color of 3, 13, and 45 nm AuNP solutions

Toxicity of AuNPs (1.4–65 nm), an inevitable issue for its biomedical applications, is related to its size, surface group, dose, and incubation time as well as cell line [33–35]. It is worth noting that 50 nm AuNPs are harmful for human OA chondrocytes [36], while 5 nm AuNPs capped with porcine type II collagen enhance chondrocytes growth in 10-kg Yorkshire pigs [37]. This report aims to study the size-dependent cytotoxicity of AuNPs in rabbit articular chondrocytes. It was reported that 18 nm AuNPs (0–500 pM) capped with citrate induced dose-dependent cytotoxicity in Hela cells [38], and we selected similar concentration (0–2 nM) to study the cytotoxicity of AuNPs in our system. The AuNPs size of our preparation is 3, 13, and 45 nm, respectively, just covering the size of 1–100 nm (Fig. 1a), and AuNPs with this size range seem to have more practical significance [15, 24, 30–32]. Our data demonstrates that 13 nm AuNPs capped with citrate induce chondrocyte apoptosis, whereas 3 and 45 nm AuNPs are nontoxic in chondrocytes.

## Methods

### Reagents

2’, 7’-Dichlorofluorescin diacetate (DCFH-DA), Nacetyl cysteine (NAC), Rhodamine 123 (Rho 123), Hoechst 33258 and HAuCl_4_ were from Sigma (St. Louis, MO, USA). Dulbecco’s Modified Eagle’s Medium (DMEM) was from Gibco (Carlsbad, CA, USA), fetal bovine serum (FBS) was from Sijiqing (Hangzhou, Zhejiang, China). Cell Counting Kit-8 (CCK-8) was from Dojindo (Kumamoto, Japan). Annexin V-FITC/propidium iodide (PI) apoptosis detection kit was from Bender Med-systems (Vienna, Austria). Mitotracker Deep Red 633, Trypsin and type II collagenase were from Invitrogen (Carlsbad, CA, USA). Sodium citrate dehydrate was purchased from Sinopharm Group Chemical Regent Co., Ltd. (Shanghai, China).

### Preparation of AuNPs

As described previously, 13 nm AuNPs were prepared by the citrate reduction of HAuCl_4_ [39]. All glass wares were cleaned in aqua regia (3 parts HCl, 1 part HNO_3_) and rinsed with MilliQ water. A solution of HAuCl_4_ (1 mM, final concentration) in MilliQ water (50 mL) in a three-necked flask with a condenser and a thermometer was heated while stirring. After boiling had commenced, trisodium citrate solution (5 mL, 38.8 mM) was added quickly, which resulted in a change in solution color from pale yellow to deep red. After the color change, the solution was kept stirring for additional 15 min and then slowly cooled down to room temperature.

It is possible to control the size of AuNPs from 5 to 150 nm by simply varying the reaction conditions [40–43]. We prepared 3 nm AuNPs by changing the ratio of sodium citrate to gold salt. Similar to the preparation of 13 nm AuNPs, aqueous solution of HAuCl_4_ (50 mL, 0.29 mM) was brought to a three-necked flask with a condenser and a thermometer while stirring and boiling, and trisodium citrate solution (4 mL, 34 mM) was added quickly after boiling had commenced, which resulted in a change in solution color from pale yellow to deep red. After the color change, we stopped boiling and kept stirring for additional 15 min to cool it to room temperature.

Although working very hard, we failed to prepare AuNPs more than 13 nm in diameter. Finally, we purchased 45 nm AuNPs from British BioCell International (BBI, Cardiff, UK, 60 nm AuNPs named by BBI), and the concentration of these particles was ~0.04 nM according to spectrophotometry measurement. Therefore, the highest concentration of 45 nm AuNPs was 0.02 nM in our system, while the highest concentration was 2 nM for both 3 and 13 nm AuNPs.

The formation of AuNPs was analyzed with a UV-vis spectrophotometer (Lambda 35, Perkin-Elmer, USA). The diameter and zeta potential of AuNPs were measured by dynamic light scattering (DLS, *n* = 3, Zetasizer Nano, Malvern Instrument Co., UK).

### Cell Isolation and Culture

Articular chondrocytes for primary culture were harvested from slices of shoulder, knee and hip-joint cartilage from 4-week-old New Zealand white rabbits under aseptic conditions as described previously [26]. The cartilage was separated from the subchondral bone and cut into small pieces (2–3 mm^2^) using a sterile surgical blade, and chondrocytes from the small pieces were then isolated by enzymatic digestion of 0.25 % Trypsin in PBS for 1 h and 0.2 % type II collagenase in DMEM for 4–6 h at 37 °C in humidified atmosphere containing 5 % CO_2_. After collection by centrifugation (4 °C, 3000 RPM, 5 min), chondrocytes were resuspended in DMEM supplemented with 10 % FBS and antibiotics (100 U/ml penicillin and 100 U/ml streptomycin), and then seeded in culture flasks at 37 °C in humidified 5 % CO_2_ as monolayer culture. The primary cells were sub-cultured to generation 2 that were cultured in DMEM supplemented with 10 % FBS and antibiotics for at least 24 h.

The second generation of chondrocytes was used for all experiments, and the cell confluence should reach approximately 80 % of the area of culture flask before experiments. Chondrocytes were treated with AuNPs for 8 h for the assay of cytochrome c release, mitochondrial morphology, and apoptosis, and for 24 h for other’s experiments without special indication.

### Characterization of Cell Death

Cell viability was detected by CCK-8 assay according to the manufacturer’s instructions as described previously [44, 45]. Cells were plated in 96-well plates (1.3 × 10^4^ cells/well) for 24 h at 37 °C and then subjected to the indicated treatments, including treatment with 3 nm AuNPs (0–2 nM), 13 nm AuNPs (0–2 nM) and 45 nm AuNPs (0–0.02 nM), respectively, for 24 h, and treatment with 13 nm AuNPs (2 nM) for 0, 3, 6, 12 and 24 h, respectively. Afterward, 90 μl DMEM and 10 μl CCK-8 solutions were added to each sample, and after incubation at 37 °C in humidified 5 % CO_2_ for 30 min, the optical density of each sample was measured at 450 nm using an auto-microplate reader (Infinite M200, Tecan, Austria). Four parallel replicates were read for each sample.

Apoptosis rate was measured by flow cytometry (FCM) analysis using annexin V/PI apoptosis detection kit as described previously [26]. After treatment with AuNPs for 8 h, the cells were washed with PBS, suspended by trypsinization, harvested by centrifugation (4 °C, 3000 RPM, 5 min) and resuspended in 400 μl binding buffer at a concentration of 9 × 10^5^ cells/ml, and then 5 μl annexin V was added into binding buffer and incubated for 15 min in dark (4 °C). Afterward, 10 μl PI was added into the above solution for 5 min in dark (4 °C) before a further addition of 600 μl PBS. Apoptosis rate was assessed by FCM analysis with annexin V/PI apoptosis detection kit, and 10,000 events were recorded for each FCM analysis.

Apoptotic morphological changes in the nuclear chromatin of cells were detected by Hoechst 33258 staining. Chondrocytes were seeded on a confocal dish. After treatment with AuNPs for 24 h, the cells were fixed with 4 % paraformaldehyde for 10 min and washed with PBS, followed by incubation with Hoechst 33258 staining (10 μM, final concentration) for 30 min in dark (37 °C). After three washes with PBS, chondrocytes stained with Hoechst 33258 were imaged by a fluorescence microscope (Axiovert 200 M, Zeiss).

### Measurement of Intracellular ROS

Intracellular ROS levels were measured with DCFH-DA as described previously [46, 47]. Chondrocytes cultured in 6-wells plates for 24 h were subjected to the indicated treatments. DCFH-DA (20 μM, final concentration) was added to different groups of chondrocytes and the cells were then incubated in a dark and humidified atmosphere (5 % CO_2_, 37 °C) for 30 min. Afterward, the cells were washed with PBS, suspended by trypsinization, harvested by centrifugation (4 °C, 3000 RPM, 5 min), resuspended in 1 ml PBS and finally analyzed by FCM with 488 nm excitation and 525 nm emission detection. Values of cellular fluorescence were obtained using FCS Express Version 3.

In addition, we studied whether ROS played a key role for the cytotoxicity induced by 13 nm AuNPs. NAC, a potent antioxidant, can prevent chondrocyte death by scavenging ROS [26, 27]. NAC was prepared just before the experiments by dissolving the powders in MilliQ water. Chondrocytes plated in 96-well plates for 24 h were pre-incubated with NAC (2 mM, final concentration) for 2 h, and then co-treated with 13 nm AuNPs (2 nM) and H_2_O_2_ (0.5 mM, final concentration), respectively, for 24 h before CCK-8 assay.

### Measurement of Mitochondrial Membrane Potential (ΔΨm)

Rho 123 was used to analyze ΔΨm by FCM as described previously [26, 46]. Cells were stained with Rho 123 (6 μM, final concentration) for 30 mins at 37 °C in dark, and then washed with PBS, suspended by trypsinization, harvested by centrifugation (4 °C, 3000 RPM, 5 min), resuspended in 1 ml PBS and subsequently assayed by FCM with 488 nm excitation and 515 nm emission. Values of cellular fluorescence were obtained using FCS Express Version 3.

The ΔΨm in single living cells was also monitored in real-time by time-lapse confocal imaging as described previously [46]. Briefly, the fluorescence images of cells stained with 6 μM Rho 123 were monitored in real-time using a confocal microscope (LSM510/ConfoCor2, Zeiss, Jena, Germany) equipped with a device sustained culture condition (37 °C, 5 % CO_2_) .

### Detection of Cytochrome c Release

Chondrocytes were transiently transfected with green fluorescent protein-cytochrome c (GFP-Cyt.c, provided by Dr. G. J. Gores) [48]. Thirty-six hours after transfection, the cells were treated with 13 nm AuNPs for 8 h, washed with PBS and imaged by the fluorescence microscope (IX73, Olympus, Japan). The GFP-Cyt.c released from mitochondria was determined based on the GFP-Cyt.c fluorescence images. GFP was excited at 488 nm and its fluorescence emission was recorded through a 500–530 nm IR band-pass filter.

### Observation of Mitochondrial Morphology

Cells were cultured on a confocal dish. After treatment with 3, 13, and 45 nm AuNPs for 8 h, the cells were washed with PBS and incubated with Mitotracker Deep Red 633 (0.1 μM, final concentration) for 30 min at room temperature in dark. The cells were then washed with PBS and visualized under confocal microscope (LSM510/ConfoCor2, Zeiss, Jena, Germany). Mitotracker Deep Red 633 was excited at 633 nm, and the emitted light was recorded through a 650-nm long-pass filter.

### Statistical Analysis

Data were presented as mean ± SD from three independent experiments, and all of experiments repeated three times. Data were analyzed by student’s *t* test using SPSS 17.0. *P* values less than 0.05 were considered to be statistically significant.

## Results

### Characterization of AuNPs

The hydrodynamic size and surface charge of AuNPs were analyzed by DLS (Fig. 1a–c). Fig. 1a shows a representative DLS analysis on the size distribution of AuNPs, and the hydrodynamic size of three diameters of AuNPs was ca. 2.94 nm (indicated as 3 nm in our text), ca. 13.19 nm (indicated as 13 nm in our text) and ca. 44.74 nm (indicated as 45 nm in our text), respectively (Fig. 1b). The corresponding zeta potential was −22.9 ± 0.3 mV for 3 nm AuNPs, −33.76 ± 0.58 mV for 13 nm AuNPs and −31.93 ± 0.41 mV for 45 nm AuNPs (Fig. 1c). The surface plasmon resonance peak of 3, 13, and 45 nm AuNPs appeared around 523, 520, and 535 nm, respectively (Fig. 1d). All of the 3, 13, and 45 nm AuNPs solutions remained red in color (Fig. 1e)**,** a characteristic of AuNPs particles less than 100 nm in diameter [49].

### 13 nm AuNPs Induce Cytotoxicity in Rabbit Articular Chondrocytes

**Fig. 2:**
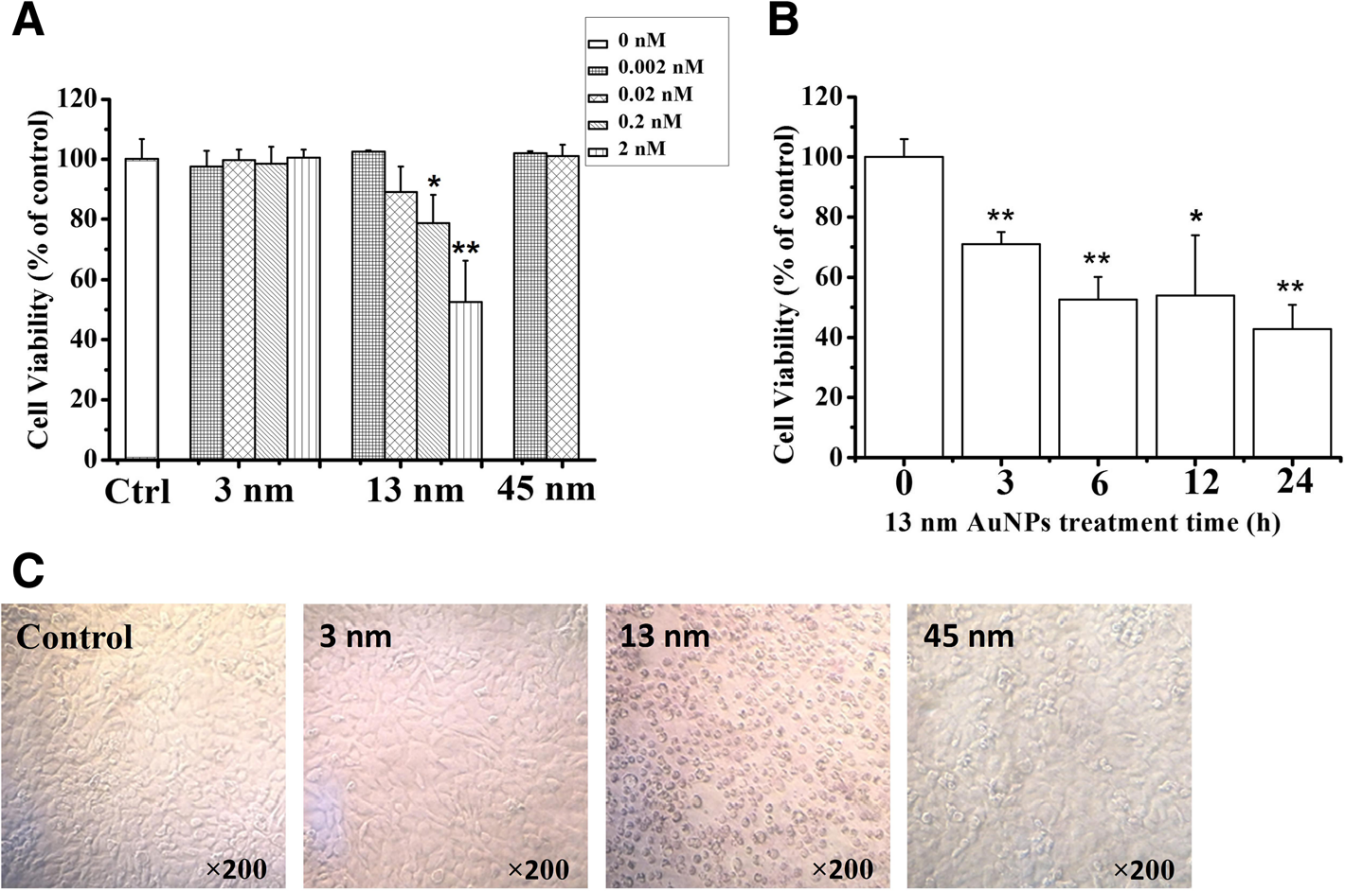
AuNPs induce cytotoxicity in rabbit articular chondrocytes. **a** cytotoxicity of 3, 13, and 45 nm AuNPs. *P < 0.05 and **P < 0.01, compared with control. **b** time-dependent cytotoxicity of 13 nm AuNPs. *P < 0.05 and **P < 0.01, compared with control. **c** Microscopic images of chondrocytes after treatment with 3, 13, and 45 nm AuNPs, respectively, for 24 h. Original magnification: ×200

CCK-8 assay showed that treatment with 13 nm AuNPs (0.02, 0.2 and 2 nM) for 24 h induced dose-dependent cytotoxicity, while treatment with 3 nm AuNPs (0.002, 0.02, 0.2, and 2 nM) or 45 nm AuNPs (0.002 and 0.02 nM) for 24 h did not induce cytotoxicity (Fig. 2a). Moreover, 13 nm AuNPs (2 nM) induced a potent time-dependent cytotoxicity (Fig. 2b). Microscopic images showed that 13 nm AuNPs (2 nM) significantly induced chondrocyte death, while 3 nm AuNPs (2 nM) and 45 nm AuNPs (0.02 nM) did not induce chondrocyte death (Fig. 2c).

### 13 nm AuNPs Induce Apoptosis in Rabbit Articular Chondrocytes

**Fig. 3:**
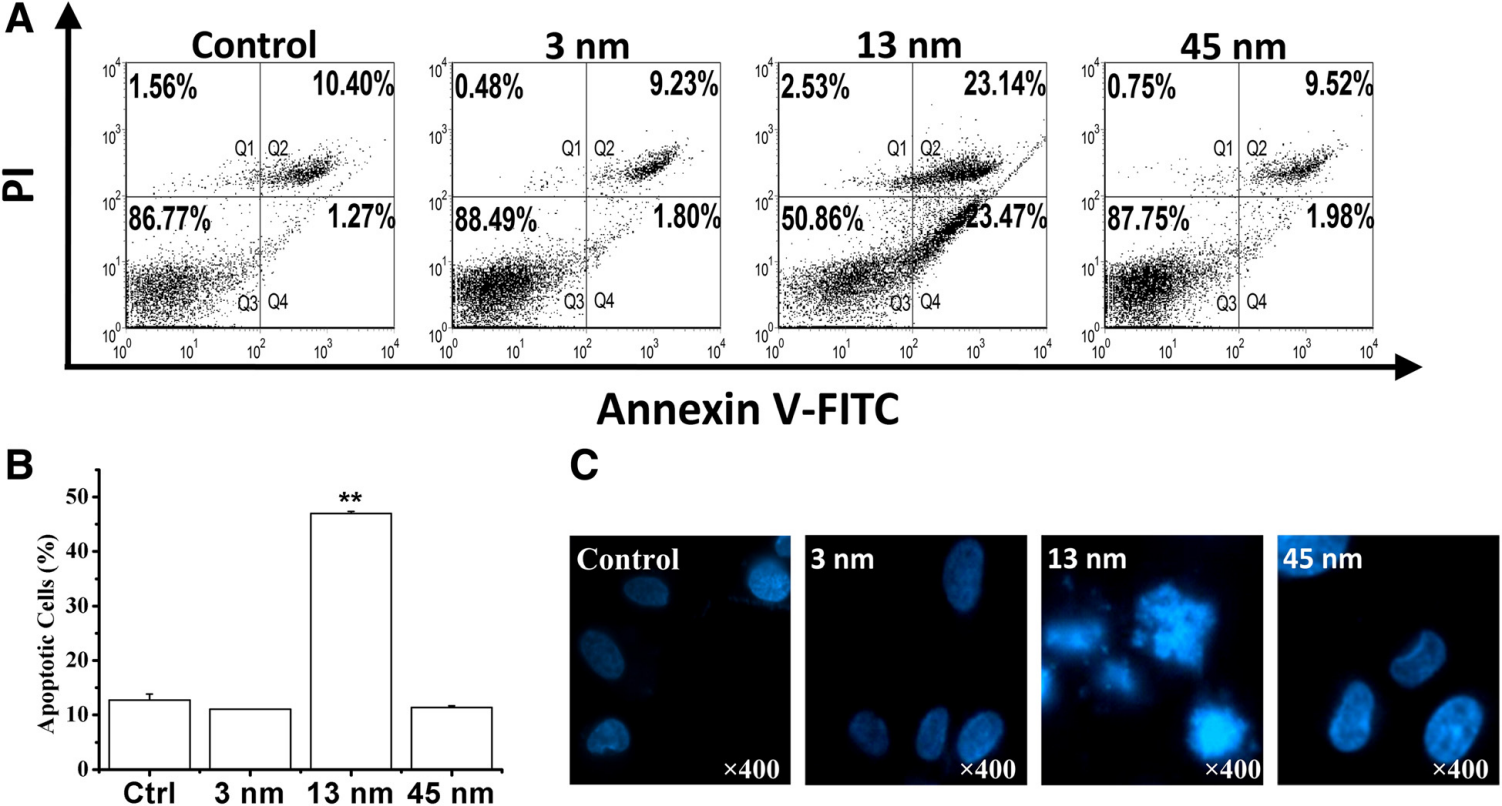
13 nm AuNPs induce apoptosis in rabbit articular chondrocytes. **a** FCM analysis with annexin V/PI staining on chondrocytes apoptosis induced by 3, 13, and 45 nm AuNPs, respectively. **b** Statistical results from three independent FCM analyses. **P < 0.01, compared with control. **c** Fluorescence imaging of chondrocytes stained with Hoechst 33258 after treatment with 3, 13, and 45 nm AuNPs, respectively, for 24 h. Original magnification: ×400

We next used FCM analysis with annexin V/PI staining to determine whether 13 nm AuNPs induced apoptosis in chondrocytes. Annexin V was used to detect phosphatidylserine externalization which was a hallmark of the early apoptosis, and PI was used to label DNA fragments, a symbol of cell death. Q3 area (both annexin V and PI are negative) represents the intact and healthy cells. Q4 (annexin V is positive and PI is negative) represents the early apoptotic cells and Q2 (both annexin V and PI are positive) represents the late apoptosis. As shown in Fig. 3a, 86.37 % cells were gathered together at Q3 area in the control group, and the percentages of apoptotic cells (Q2 + Q4) in the control, 3 and 45 nm AuNPs groups were less than 12 %. However, after treatment with 13 nm AuNPs (2 nM) for 8 h, the percentage of apoptotic cells (Q2 + Q4) increased to 46.61 %, demonstrating that 13 nm AuNPs induced chondrocyte death in apoptotic fashion, which was further verified by the fluorescence images of chondrocytes stained with Hoechst 33258 (Fig. 3c).

### 13 nm AuNPs Induce Apoptosis via Mitochondrial Damage

**Fig. 4:**
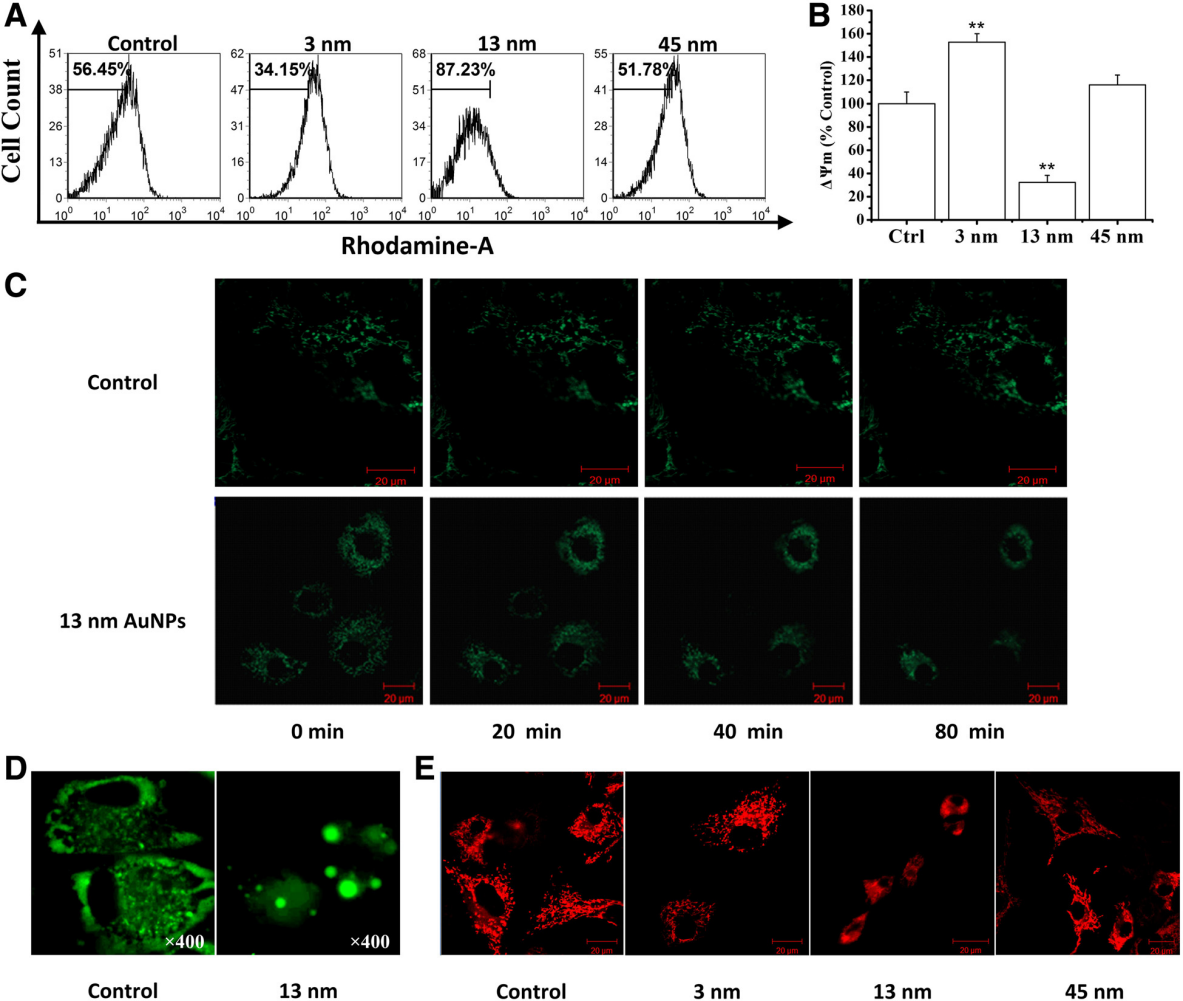
13 nm AuNPs induce apoptosis by mitochondrial damage. **a** FCM analysis with Rho 123 staining on the loss of ΔΨm induced by 3, 13, and 45 nm AuNPs, respectively. **b** Statistical results from three independent FCM analyses. **P < 0.01, compared with control. **c** Time-lapse imaging of cells stained with Rho 123 after treatment with 13 nm AuNPs for 8 h. *Scale bar* 20 μm. **d** Fluorescence imaging of chondrocytes transfected with GFP-Cyt.c after treatment with 13 nm AuNPs for 8 h. Original magnification: ×400. **e** Confocal imaging of chondrocytes stained with Mitotracker Red 633 after treatment with 3, 13, and 45 nm AuNPs, respectively, for 8 h. *Scale bar* 20 μm

FCM analysis with Rho 123 staining showed that 13 nm AuNPs induced a significant decrease of ΔΨm, while 3 and 45 nm AuNPs did not induce a decrease of ΔΨm (Fig. 4a, b). Dynamical loss of ΔΨm in single living cells stained with potential-sensitive dye Rho 123 was also monitored by imaging the reduction of Rho 123 fluorescence using a time-lapse confocal microscope. The time-lapse images of cells stained with Rho 123 are shown in Fig. 4c. We found that compared with control cells, 13 nm AuNPs treatment induced a significant decrease of Rho 123 fluorescence, further confirming the notion that 13 nm AuNPs induce chondrocyte apoptosis via mitochondrial damage.

We next monitored cytochrome c release from mitochondria in single living cells using the fluorescence microscope. As shown in Fig. 4d, in contrast to control cells, the cells treated with 13 nm AuNPs exhibited an even distribution of the GFP-Cyt.c in the entire cytoplasm, indicating that 13 nm AuNPs induced the release of cytochrome c from mitochondria. We also assessed the effect of AuNPs on the mitochondrial morphology by using confocal fluorescence microscope, and found that in contrast to the normal long tubular mitochondria in control cells, the mitochondria of the cells treated with 13 nm AuNPs became punctate and swollen (Fig. 4e), further demonstrating the notion that 13 nm AuNPs induced damage of mitochondria.

### 13 nm AuNPs Induce Cytotoxicity Independent of ROS

**Fig. 5:**
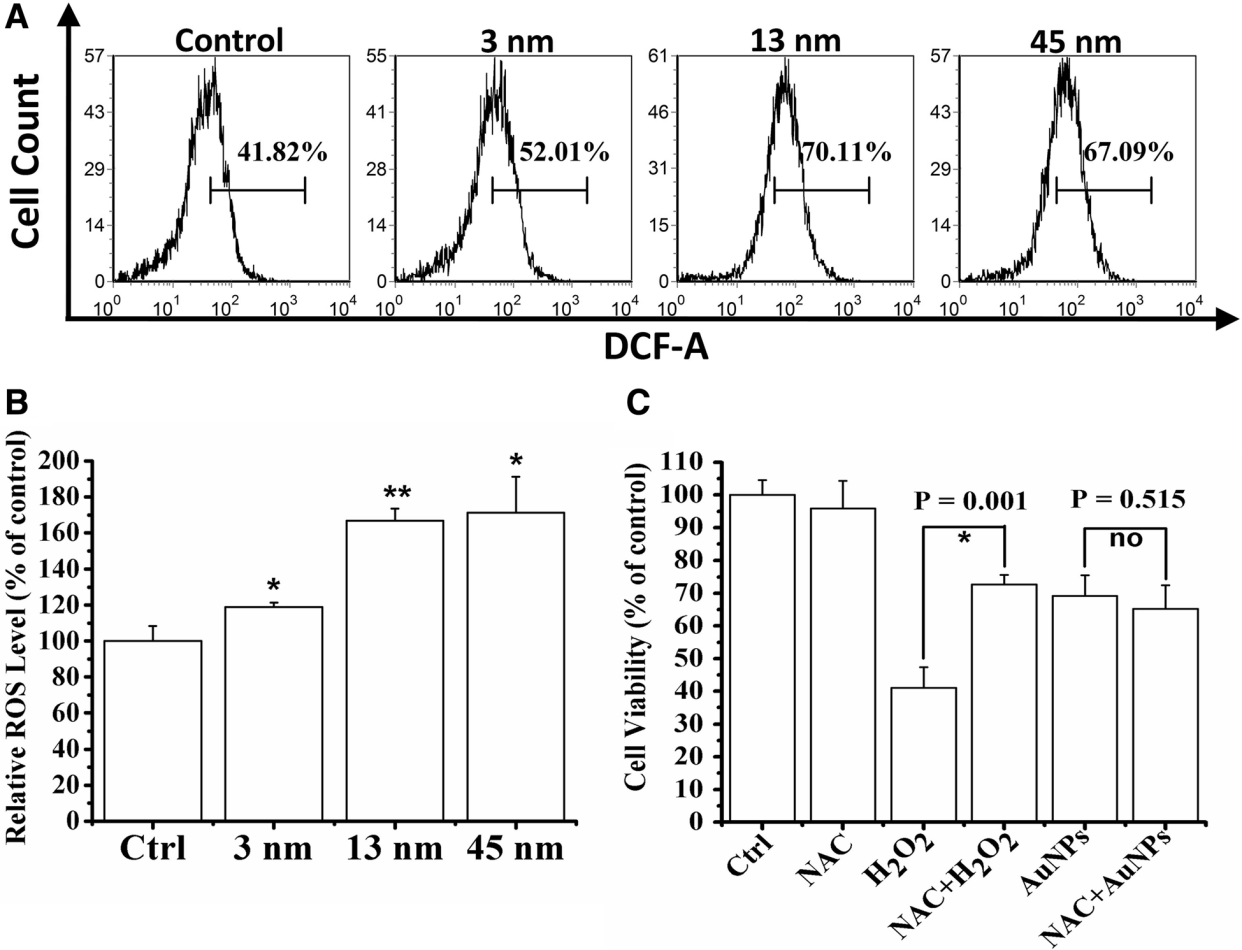
13 nm AuNPs induce cytotoxicity independent of ROS. **a** FCM analysis on the ROS generation induced by 3, 13, and 45 nm AuNPs, respectively. **b** Statistical results from three independent FCM analyses. *P < 0.05 and **P < 0.01, compared with control. **c** Effect of NAC pretreatment on the cytotoxicity of 13 nm AuNPs

Finally, we examined whether ROS was involved in the cytotoxicity of 13 nm AuNPs. We firstly used FCM assay with DCFH-DA to evaluate AuNPs-induced ROS production, and found that treatment with three diameters of AuNPs for 24 h induced a significant ROS generation (Fig. 5a, b). CCK-8 assay showed that NAC pretreatment prevented 500 μM H_2_O_2_-induced cell death but did not prevent 13 nm AuNPs-induced cytotoxicity (Fig. 5c), demonstrating that 13 nm AuNPs induced cytotoxicity possibly independent of ROS.

## Discussion

Our results support the notion that AuNPs induce size- and dose-dependent cytotoxicity in chondrocytes. Furthermore, this report for the first time demonstrates that 13 nm AuNPs capped with citrate induce chondrocyte apoptosis by the mitochondrial pathway. Although AuNPs induce ROS generation, the ROS may be not involved in the cytotoxicity of AuNPs.

Our findings that 13 nm AuNPs induce chondrocyte apoptosis but 3 and 45 nm AuNPs are nontoxic (Figs. 2a and 3) demonstrate the size-dependent toxicity of AuNPs in chondrocytes. We noted that 1.2 nm AuNPs capped with triphenylphosphine monosulfonate (TPPMS) rather than 15-nm particles induced apoptosis in Hela cells [35], whereas larger AuNPs (25 nm), when capping with polyvinyl pyrrolidone, caused more cytotoxicity in Hela cells compared to smaller particles (2 and 10 nm) [34]. Similarly, in vivo studies in mice also showed different size-dependent toxicity of AuNPs capped with different surface groups (citrate or polyethylene glycol) [50, 51]. The different size-dependent toxicity in the same cell line or animal may be related to the different surface groups of AuNPs. Although 13 nm AuNPs capped with citrate caused cytotoxicity in rabbit articular chondrocytes (Fig. 2), 10 nm AuNPs were reported to be nontoxic in dendritic cells [52]. In addition, 33 nm AuNPs capped with citrate were found to be non-cytotoxic to baby hamster kidney cells and human hepatocellular liver carcinoma cells, but cytotoxic to a human carcinoma lung cell line [53]. Therefore, the size-dependent cytotoxicity of AuNPs is also related to cell line.

Many drugs that are beneficial at low doses are toxic at high doses [33]. Similarly, 1.2 or 1.4 nm AuNPs at high doses showed dose-dependent cytotoxicity in vitro study [35, 54]. Further, 13 nm AuNPs at low dose (0.2–2 nM) showed dose-dependent cytotoxicity in rabbit articular chondrocytes (Fig. 2a). It was reported that 50 nm AuNPs less than 80 μM were found to be non-cytotoxic, while 160 μM of 50 nm AuNPs was harmful for human OA chondrocytes [36]. Consequently, we conclude that 13 nm AuNPs are more toxic than 50 nm AuNPs in chondrocytes.

Our data demonstrate that 13 nm AuNPs induce cell death by mitochondrial damage (Fig. 4), which is consistent with Pan and coworkers’ report [54]. Similarly, in contrast to 2 and 10 nm AuNPs, it was reported that 25 nm AuNPs aggregated in the mitochondria of HeLa cells and caused cytotoxicity [34]. Recently, Mkandawire and coworkers’ reported that 20 nm AuNPs targeted into mitochondria of breast cancer cells, caused partial rupture of the outer mitochondrial membrane and finally induced apoptosis [55]. These findings proved that mitochondrial damage induced by AuNPs played a crucial role in cell death. Interestingly, 3 nm AuNPs significantly enhanced the proportion of cells with high mitochondrial membrane potential (Fig. 4a, b). Dwivedi and coworkers found that 13 nm AuNPs combined with chondroitin sulfate induced the growth of chondrocytes and also enhanced the production of extracellular matrix components [24]. These data suggest that AuNPs with specific size have the potential ability to trigger chondrocyte growth.

Formation and degradation of the extracellular matrix are important for cartilage. Extracellular matrixes, including collagens, proteoglycans, and noncollagenous proteins, play a key role in forming a macromolecular framework to stabilize tissue [56]. Compared to chondrocytes grown in monolayer culture (two-dimensional structure), animal experiments (three-dimensional structure) may be more suitable for the study of extracellular matrix. Therefore, we are going to evaluate the effect of AuNPs on the extracellular matrix formation and degradation in mice OA model in the near future.

Although 13 nm AuNPs increased ROS product in chondrocytes (Fig. 5a, b), the fact that NAC pretreatment did not prevent its cytotoxicity (Fig. 5c) suggests that 13 nm AuNPs induce ROS-independent cytotoxicity. Similarly, Tay and coworkers found that ~2 nm Au nanoclusters capped with mercaptopropionic acid increased ROS product in both mitochondria and cytoplasm, but it did not lead to any detrimental cellular effects in human derived colonic epithelial NCM460 cells [57]. In fact, the effect of AuNPs on ROS production is still controversial. Inhibiting ROS production was considered as a remarkable property of AuNPs [29, 49]. Hsieh and coworkers found that compared to epigallocatechin-3-gallate (EGCG), ~50 nm EGCG-AuNPs conjugates had stronger ability to scavenge ROS [28]. Similar result was also reported by another study on the articular chondrocytes of 10-kg Yorkshire pigs [37]. However, there was publication reported that AuNPs could significantly induce surface group-dependent intracellular ROS generation [58]. It was reported that 1.4 nm AuNPs capped with TPPMS triggered necrosis by oxidative stress in Hela cells [54]. Therefore, the role of ROS in AuNPs-induced cytotoxicity may depend on cell lines and the surface groups of AuNPs.

Although 3 and 13 nm AuNPs were prepared by the same method, the two particles had different zeta potential values (Fig. 1c), further demonstrating the notion that the zeta potential value of nanoparticles is associated with the size of the particles. It was reported that zeta potential values were related to particles’ electrophoretic mobility [59]. In addition, previous studies indicated that the electrophoretic mobility of colloidal particles (in the same medium) was related to the size of particles, and that the electrophoresis mobility increased with the increasing radius of colloid particles, which might be due to the relaxation effect of smaller particles [60].

## Conclusions

While 3 and 45 nm AuNPs are nontoxic, 13 nm AuNPs induce cytotoxicity in rabbit articular chondrocytes. Moreover, 13 nm AuNPs induce chondrocyte apoptosis via mitochondrial damage independent of ROS. Our findings further remind those who use AuNPs for treatment of OA should pay attention to the size-dependent cytotoxicity of AuNPs in chondrocytes.

## Abbreviations

AuNPs: gold nanoparticles
CCK-8: Cell Counting Kit-8
CIA: collagen-induced arthritis
DCFH-DA: 2’, 7’-dichlorofluorescin diacetate
DLS: dynamic light scattering
DMEM: Dulbecco’s Modified Eagle’s Medium
EGCG: epigallocatechin-3-gallate
FBS: fetal bovine serum
FCM: flow cytometry
GFP-Cyt.c: green fluorescent protein-cytochrome c
NAC: nacetyl cysteine
NSAIDs: non-steroidal anti-inflammatory drugs
OA: osteoarthritis
PI: propidium iodide
Rho 123: rhodamine 123
ROS: reactive oxygen species
TPPMS: triphenylphosphine monosulfonate
ΔΨm: mitochondrial membrane potential
